# Synthesis, characterization, and lipoxygenase inhibition of salicylaldehyde-derived schiff base metal complexes:  enzymatic and in silico evaluation using quinoa lipoxygenase

**DOI:** 10.1007/s00210-025-04955-6

**Published:** 2026-01-16

**Authors:** Ergün Ereminsoy, Yeliz Demir, Sümeyra Tuna Yıldırım, Cüneyt Türkeş, Ömer İrfan Küfrevioğlu

**Affiliations:** 1 Department of Chemistry, Faculty of Science, Atatürk University, Erzurum, 25240 Türkiye; 2 Department of Pharmacy Services, Nihat Delibalta Göle Vocational High School, Ardahan University, Ardahan, 75700 Türkiye; 3 Department of Analytical Chemistry, Faculty of Pharmacy, Erzincan Binali Yıldırım University, Erzincan, 24002 Türkiye; 4 Department of Biochemistry, Faculty of Pharmacy, Erzincan Binali Yıldırım University, Erzincan, 24002 Türkiye

**Keywords:** Metal complexes, Characterization, Lipoxygenase, Molecular docking, Schiff base

## Abstract

In the present study, novel Schiff base ligands were synthesized via the condensation of salicylaldehyde derivatives with *o*-aminophenol, and their metal complexes with Co(II), Ni(II), Cu(II), Zn(II), Cd(II), and UO₂(VI) metal actetates were successfully prepared. The compounds were comprehensively characterized using FT-IR, ^1^H-NMR, ^13^C-NMR, UV–Vis spectroscopy, X-ray diffraction (XRD), scanning electron microscopy (SEM), and magnetic susceptibility measurements. FT-IR spectra confirmed azomethine (C = N) coordination through characteristic bands at 1612–1630 cm^−1^, while ^1^H-NMR spectra exhibited diagnostic azomethine proton signals at δ 8.84–8.62 ppm. XRD analysis revealed a semi-crystalline nature with prominent reflections at 2θ = 18.02° and 20.87°, and SEM images demonstrated nanorod-like morphologies. Magnetic moment values supported the proposed coordination geometries of the paramagnetic complexes. The biological activity of the synthesized compounds was investigated through in vitro inhibition studies against lipoxygenase (LOX), a ~ 97 kDa enzyme purified from Chenopodium quinoa Willd. with 77.89% purity and 7.24% yield. All compounds exhibited competitive inhibition toward LOX, with inhibition constants (K_i_) in the range of 0.014 ± 0.002 to 0.858 ± 0.194 μM, indicating remarkable inhibitory potency. Molecular docking studies further substantiated the experimental results by revealing key hydrogen-bonding and π-cation interactions between the most active compounds and essential amino acid residues within the LOX active site. Overall, the results demonstrate that these Schiff base metal complexes represent promising candidates for the development of potent LOX inhibitors.

**Supplementary Information:**

The online version contains supplementary material available at 10.1007/s00210-025-04955-6.

## Introduction

Metalloenzymes are a broad class of enzymes that rely on one or more metal ions such as Fe^2^⁺, Zn^2^⁺, Cu^2^⁺ or Mn^2^⁺ to maintain structural stability or to participate directly in catalysis. These metal ions can mediate redox processes, stabilize transition states, or facilitate substrate binding, thereby playing a crucial role in various biochemical reactions (Smith 2023). Among them, lipoxygenases (LOXs) are non-heme iron-containing metalloenzymes that catalyze the dioxygenation of polyunsaturated fatty acids into hydroperoxides. These enzymes are involved in a range of physiological and pathological processes, including inflammation, cancer progression, and neurodegenerative disorders (Bhuktar et al. 2023). LOX amplifies inflammation via the synthesis of the inflammatory mediators known as leukotrienes, has been shown to have a role in the onset of diabetic neuropathies (Dobrian et al. 2019). Nonsteroidal anti-inflammatory medicines including oxaprozin, meloxicam, naproxen, and nabumetone have been shown to diminish inflammatory processes by blocking the LOX enzyme (Bindu et al. 2020). The health complications associated with clinically approved anti-diabetic and anti-inflammatory medications, including issues in the hematologic systems, hepatic, cardiovascular, gastric mucosa, and renal have prompted interest in the development of LOX inhibitors that exhibit minimal or no side effects in drug discovery (Martel-Pelletier et al. 2003). Despite their biological significance, currently available LOX inhibitors often suffer from poor selectivity, low bioavailability, or unwanted side effects, limiting their clinical potential (Rudrapal et al. 2023). Therefore, the development of novel and more efficient LOX inhibitors remains a relevant and urgent goal.

Schiff bases are often used ligands owing to their biological and structural attributes; therefore, their significance is on the rise (Savcı et al. 2022; Buldurun et al. 2020, 2022a). Schiff bases provide a versatile platform for structural modification via the azomethine (-HC = N-) moiety, allowing the substitution of R and/or R' groups with diverse pharmacophores (Buldurun et al. 2022b; Alkış et al. 2023). This enables fine-tuning of electronic properties, lipophilicity, and spatial orientation, which can significantly influence biological interactions (Shebl 2016). Upon coordination with metal ions, the resulting metal-Schiff base complexes acquire additional redox-active centers and steric attributes, often enhancing their bioavailability, enzyme affinity, and overall pharmacological potential (Adly et al. 2017). It is also known that some metal Schiff base complexes have antifungal (Mandal et al. 2024), antibacterial (Yousif et al. 2017), antimicrobial (Dinku et al. 2024), anti-inflammatory (Bufarwa et al. 2025) anticancer (Sanjurani et al. 2024), and antitumor (Iacopetta et al. 2021) properties. Given their diverse biological activities and strong chelating potential, metal Schiff base complexes have drawn significant attention in drug design and development studies.

Beyond bioactivity, Schiff bases have substantial value in analytical and optical applications. Owing to strong coordination ability and tunable intramolecular charge transfer, Schiff base derivatives are extensively developed as chemosensors and fluorescent probes for metal ions and other analytes, with modern designs leveraging mechanisms such as PET/ICT, aggregation-induced emission, and environment-sensitive fluorescence responses. These features make Schiff bases particularly attractive as compact, designable platforms for selective detection, imaging, and signal transduction (Abdel Halim and Shebl 2022; Elabeden et al. 2025; Taha et al. 2017; Shebl et al. 2016).

Despite extensive studies on LOX inhibition, previously published work has largely focused on either the biological evaluation of natural or synthetic inhibitors using commercially available LOX enzymes, or on the synthesis and characterization of Schiff base metal complexes without direct integration into a purified enzymatic system. Moreover, most reported LOX inhibitors exhibit inhibitory activity in the micromolar range, and studies combining enzyme purification from alternative plant sources with metal-assisted inhibitor design remain limited. In contrast, the present study uniquely integrates (i) purification of lipoxygenase from an underexplored plant source (*Chenopodium quinoa*), (ii) rational design and synthesis of salicylaldehyde-derived Schiff base ligands and their metal complexes, and (iii) comprehensive kinetic and in silico analyses to elucidate structure–activity relationships. This combined enzymological and coordination chemistry approach distinguishes the present work from previous studies and enables the identification of metal–Schiff base complexes with submicromolar and nanomolar-level LOX inhibitory potency, thereby advancing current strategies for LOX inhibitor development.

## Results and discussion

### Structural characterization

The coordination of L^1^H and L^2^H with Co^2+^, Ni^2+^, Cu^2+^, Zn^2+^, Cd^2+^ and UO_2_^2+^ stoichiometric ratio, and stereochemistry of ligands and metal complexes were investigated based upon various techniques. The ligands and their corresponding metal complexes were obtained in satisfactory yields. As is seen in Tables 1 and 2, it was determined that the metal:ligand ratio is 1:2 in the elemental analysis of the metal complexes of the ligands. Some analytical and physical data of the synthesized compounds were given in Tables 1 and 2.

**Table 1:** Analytical and physical data of L^1^H and its metal complexes

Compound	Formula	MW (g/mol)	Colour	M.p (^o^C)	μ_eff_	Yield (%)	Elemental analysis, % calculated (found)		
							C	H	N
L^1^H	C_13_H_11_NO_3_	229.235	Red	216–218	-	88.00	68.11 (68.09)	4.84 (4.79)	6.11 (6.02)
[Co(L^1^)_2_]	CoC_26_H_20_N_2_O_6_	515.387	Dark Brown	> 300	4.21	62.00	60.59 (60.46)	3.91 (3.86)	5.43 (5.39)
[Ni(L^1^)_2_]	NiC_26_H_20_N_2_O_6_	515.147	Brown	> 300	2.71	54.00	60.62 (60.59)	3.91 (3.86)	5.44 (5.39)
[Cu(L^1^)_2_]	CuC_26_H_20_N_2_O_6_	520.000	Dark Green	> 300	1.84	63.00	60.05 (60.01)	3.88 (3.78)	5.39 (5.28)
[Zn(L^1^)_2_]	ZnC_26_H_20_N_2_O_6_	521.844	Light Green	> 300	Diamagnetic	65.00	59.84 (59.72)	3.86 (3.79)	5.37 (5.29)
[Cd(L^1^)_2_]	CdC_26_H_20_N_2_O_6_	568.864	Light Green	> 300	Diamagnetic	52.00	54.90 (54.82)	3.54 (3.46)	4.92 (4.89)
[UO_2_(L^1^)_2_]	UC_26_H_20_N_2_O_8_	726.482	Dark Brick	> 300	Diamagnetic	71.00	42.99 (42.86)	2.77 (2.69)	3.86 (3.79)

**Table 2:** Analytical and physical data of L^2^H and its metal complexes

Compound	Formula	MW (g/mol)	Colour	M.p (^o^C)	μ_eff_	Yield (%)	Elemental analysis, % calculated (found)		
							C	H	N
L^2^H	C_13_H_10_N_2_O_4_	258.233	Dark Melon	206–208	-	81.00	60.46 (60.32)	3.90 (3.86)	10.85 (10.72)
[Co(L^2^)_2_]	CoC_26_H_18_N_4_O_8_	573.383	Dark Brown	> 300	4.51	61.00	54.46 (54.48)	3.16 (3.08)	9.77 (9.68)
[Ni(L^2^)_2_]	NiC_26_H_18_N_4_O_8_	573.143	Light Brown	> 300	2.69	52.00	54.49 (54.37)	3.16 (3.18)	9.77 (9.63)
[Cu(L^2^)_2_]	CuC_26_H_18_N_4_O_8_	577.996	Dark Green	> 300	1.82	61.00	54.03 (54.06)	3.14 (3.06)	9.69 (9.58)
[Zn(L^2^)_2_]	ZnC_26_H_18_N_4_O_8_	579.840	Yellow	> 300	Diamagnetic	64.00	53.86 (53.69)	3.13 (3.07)	9.66 (9.58)
[Cd(L^2^)_2_]	CdC_26_H_18_N_4_O_8_	626.860	Light Yellow	> 300	Diamagnetic	56.00	49.82 (49.80)	2.89 (2.71)	8.94 (8.78)
[UO_2_(L^2^)_2_]	UC_26_H_18_N_4_O_10_	784.478	Dark Brick	> 300	Diamagnetic	69.00	39.81 (39.78)	2.31 (2.27)	7.14 (7.08)

The IR spectra of the ligands (L^1^H and L^2^H) and their corresponding metal complexes provide compelling evidence for the proposed coordination mode through systematic changes in characteristic vibrational bands. In the spectra of the free ligands, the azomethine (C = N) stretching vibrations appear at 1612 cm^−1^ for L^1^H and 1630 cm^−1^ for L^2^H, which are within the typical range reported for Schiff base ligands. Upon coordination, these bands shift to lower wavenumbers in the range of 1604–1625 cm^−1^. This shift is attributed to a decrease in the C = N bond order caused by donation of the azomethine nitrogen lone pair to the metal ion, a phenomenon well documented for metal–Schiff base complexes (Ahmed et al. 2013; Singh et al. 2017).

The broad phenolic O–H stretching bands observed at 3430 cm^−1^ (L^1^H) and 3435 cm^−1^ (L^2^H) in the free ligands (Fig. S1) are significantly altered in the spectra of the metal complexes. The weakening or disappearance of these bands upon complexation indicates deprotonation of the phenolic hydroxyl group, followed by coordination through the oxygen atom. This behavior is characteristic of phenolate-type coordination and strongly supports the involvement of the phenolic oxygen in metal binding. Further support for this coordination mode is provided by shifts in the C–O stretching vibrations, which appear at 1272 cm^−1^ (L^1^H) and 1294 cm^−1^ (L^2^H) in the free ligands. The slight shifts of these bands in the metal complexes reflect changes in the C–O bond environment due to coordination via the deprotonated phenolic oxygen atom. Importantly, new bands appear in the low-frequency region (400–600 cm^−1^) in the spectra of the metal complexes that are absent in the free ligands. These bands can be reasonably assigned to metal–nitrogen (M–N) and metal–oxygen (M–O) stretching vibrations. The bands observed in the 420–480 cm^−1^ region are attributed to M–N stretching modes, while those appearing in the 500–560 cm^−1^ region are assigned to M–O vibrations, consistent with literature reports for transition metal complexes containing N,O-donor Schiff base ligands. The presence of these coordination-induced bands provides direct spectroscopic evidence for metal–ligand bond formation. The combined shifts in the C = N, O–H, and C–O stretching vibrations, together with the emergence of characteristic M–N and M–O bands in the low-frequency region, confirm that the ligands coordinate to the metal ions in a bidentate fashion through the azomethine nitrogen and deprotonated phenolic oxygen atoms, leading to the formation of stable chelate complexes.

In the ^1^H-NMR spectra of the free ligands, the azomethine proton (–CH = N–) appears as a singlet at 8.84 ppm for L^1^H and at 8.62 ppm for L^2^H, confirming the successful formation of the Schiff base framework. These chemical shift values fall within the characteristic range reported for azomethine protons in related Schif f base systems and reflect the deshielded nature of the imine proton due to the adjacent electronegative nitrogen atom. The phenolic OH proton resonates as a sharp singlet at 12.89 ppm in L^1^H and at 15.74 ppm in L^2^H. The pronounced downfield position of these signals is indicative of strong intramolecular hydrogen bonding between the phenolic OH group and the azomethine nitrogen, a common feature in ortho-hydroxy Schiff bases. The more downfield shift observed for L^2^H can be rationalized by differences in electronic effects arising from substituent-induced variations in electron density, which enhance hydrogen bond strength and further deshield the phenolic proton. The aromatic proton signals appear as multiplets in the range of 6.76–7.34 ppm for L^1^H and 6.68–8.38 ppm for L^2^H (Fig. S2). The observed chemical shift distribution and integration patterns are consistent with those reported for structurally related Schiff base ligands (Latha et al. 2022; Rajimon et al. 2023), supporting the proposed ligand structures. Variations in the aromatic proton chemical shifts between L^1^H and L^2^H are attributed to differences in substituent effects and conjugation, which influence the electronic environment of the aromatic rings.

The ^13^C-NMR spectrum of L^1^H recorded in DMSO-d_6_ exhibited a characteristic resonance for the imine carbon at δ = 162.0 ppm. The signal corresponding to the C–OH carbon was observed at δ = 153.8 ppm, whereas the C–N carbon appeared at δ = 135.9 ppm. The aromatic ring carbons resonated within the range of δ = 116.9–128.3 ppm. The ^13^C NMR spectrum of L^2^H recorded in DMSO-d_6_ displayed the imine carbon signal at δ = 164.0 ppm. The resonance attributed to the C–OH carbon was detected at δ = 159.1 ppm, while the C–N carbon signal appeared at δ = 141.3 ppm. The aromatic ring carbon signals were observed in the range of δ = 117.1–131.4 ppm (Fig. S3). These chemical shift variations suggest that the electronic environments around the imine, hydroxyl, and amine functional groups are slightly different in the two ligands, likely due to differences in substituent effects or intramolecular interactions (Latha et al. 2022; Rajimon et al. 2023).

Although the Zn(II), Cd(II), and UO_2_(VI) complexes are diamagnetic in nature and, in principle, suitable for routine ^1^H and ^13^C-NMR analysis, reliable NMR spectra of these complexes could not be obtained due to their limited solubility and/or incomplete dissolution in common deuterated solvents (such as DMSO-d_6_ and CDCl_3_). Attempts to improve solubility resulted in either very weak signals or severely broadened and unresolved resonances, precluding meaningful spectral interpretation. Consequently, NMR analysis was restricted to the free ligands, while coordination in the metal complexes was corroborated using complementary spectroscopic techniques, including FTIR, UV–Vis., magnetic susceptibility, and elemental analysis.

XRD patterns of the Schiff base ligand was obtained at room temperature using CuKα1 λ = 1.5405 Å in the range 2θ = 10–100°, operated at 45 kV and 40 mA (Fig. S4). Diffractogram data showed that two characteristic reflection peaks belonging to the crystal structure of the ligand were clearly observed and these peaks played a distinctive role in identifying the ligand. These peaks appearing at 2θ = 18.06˚and 21.24˚, 2θ = 18.02˚ and 20.87˚ due to imine group. The XRD pattern of ligand were showed a semicrystalline nature (Khan et al. 2013; Deswal et al. 2022). The surface morphology of the ligand was investigated using SEM analysis and the ligand had a compact and dense surface morphology (Fig. S4). Also, ligand had nanorod structure and localized clusters on the surface morphology. This structure was consistent with the existing findings in the literature (Khan et al. 2013; Deswal et al. 2022).

Magnetic susceptibility measurements indicate that the Co(II), Ni(II), and Cu(II) complexes exhibit paramagnetic behavior, whereas the Zn(II), Cd(II), and UO₂(VI) complexes are diamagnetic, as expected from their electronic configurations. The effective magnetic moment (µ_eff_) values obtained for the Co(II) complexes are 4.09 B.M. and 4.51 B.M., which are consistent with the presence of three unpaired electrons corresponding to a high-spin d⁷ configuration. Such µ_eff_ values fall within the range commonly reported for Co(II) complexes in tetrahedral or distorted octahedral environments. On the basis of the magnetic data alone, the Co(II) complexes are therefore suggested to adopt a high-spin geometry, although definitive structural assignment requires complementary techniques. For the Ni(II) complexes, the measured µ_eff_ values of 2.71 B.M. and 2.69 B.M. indicate the presence of two unpaired electrons. While square-planar Ni(II), d^8^ electronic configuration, complexes are typically diamagnetic, paramagnetic behavior is well documented for Ni(II) in octahedral or pseudo-tetrahedral coordination environments. Accordingly, the observed magnetic moments suggest that the Ni(II) complexes do not possess square-planar geometry, but rather a geometry allowing unpaired electrons, such as tetrahedral or distorted octahedral coordination. However, magnetic susceptibility data alone are insufficient to unequivocally distinguish between these possibilities. The Cu(II) complexes exhibit µ_eff_ values of 1.70 B.M. and 1.82 B.M., consistent with one unpaired electron expected for d⁹ electronic configuration. These values are typical for Cu(II) complexes and support paramagnetic behavior without allowing definitive assignment of coordination geometry. As expected, the Zn(II), Cd(II), and UO_2_(VI) complexes are diamagnetic due to their closed-shell d and f electronic configurations. While their diamagnetism is consistent with filled subshells, magnetic data alone do not permit reliable determination of coordination geometry. Therefore, any proposed structural arrangement for these complexes is based on ligand denticity and complementary spectroscopic evidence rather than magnetic measurements. Overall, the magnetic susceptibility results provide supportive information regarding the electronic configurations of the metal centers and exclude square-planar geometry for the Ni(II) complexes; however, they are interpreted cautiously and in conjunction with other spectroscopic techniques.

The electronic absorption spectra of the ligands L^1^H and L^2^H were recorded in DMSO solution to investigate their electronic structures and coordination behavior. The spectra of the free ligands exhibit intense absorption bands at approximately 294 nm for L^1^H and 304 nm for L^2^H, which are assigned to π → π* transitions originating from the aromatic rings and the conjugated azomethine (–CH = N–) chromophore. Such transitions are characteristic of Schiff base systems containing extended π-conjugation. In addition to these bands, the ligands show relatively weaker and broader absorption bands at higher wavelengths, observed around 396 nm for L^1^H and 389 nm for L^2^H. These bands are attributed to n → π* transitions arising from the non-bonding lone pair electrons on the azomethine nitrogen atom. The presence of these transitions is typical for Schiff bases and reflects the availability of the imine nitrogen lone pair for potential coordination with metal ions. Upon complexation, noticeable changes are observed in the electronic spectra. The intensity of the ligand-centered π → π* and n → π* bands decreases in the metal complexes compared to the free ligands, indicating involvement of the azomethine nitrogen in coordination and a redistribution of electron density within the ligand framework. Furthermore, the n → π* transition bands undergo a bathochromic shift and appear in the range of approximately 375–435 nm in the complexes. This shift can be attributed to coordination of the azomethine nitrogen to the metal center, which reduces the availability of the lone pair electrons and alters the energy gap between the n and π* orbitals. The observed shift of the n → π* transition upon complex formation provides strong evidence for interaction between the –CH = N– group and the metal ions. Similar coordination-induced shifts of azomethine-related transitions have been widely reported for Schiff base metal complexes in the literature.

In addition, the electronic spectra of the metal complexes display new absorption bands in the 410–470 nm region, which are absent in the free ligands. These bands can be reasonably assigned to ligand-to-metal charge transfer (LMCT) transitions, originating from electron donation from the coordinated azomethine nitrogen and/or phenolate oxygen atoms to the metal-centered orbitals. The presence of these charge transfer bands further supports the formation of metal–ligand coordination bonds and confirms successful complexation (Fig. S6).

### Enzyme optimization and purification studies

**Fig. 1:**
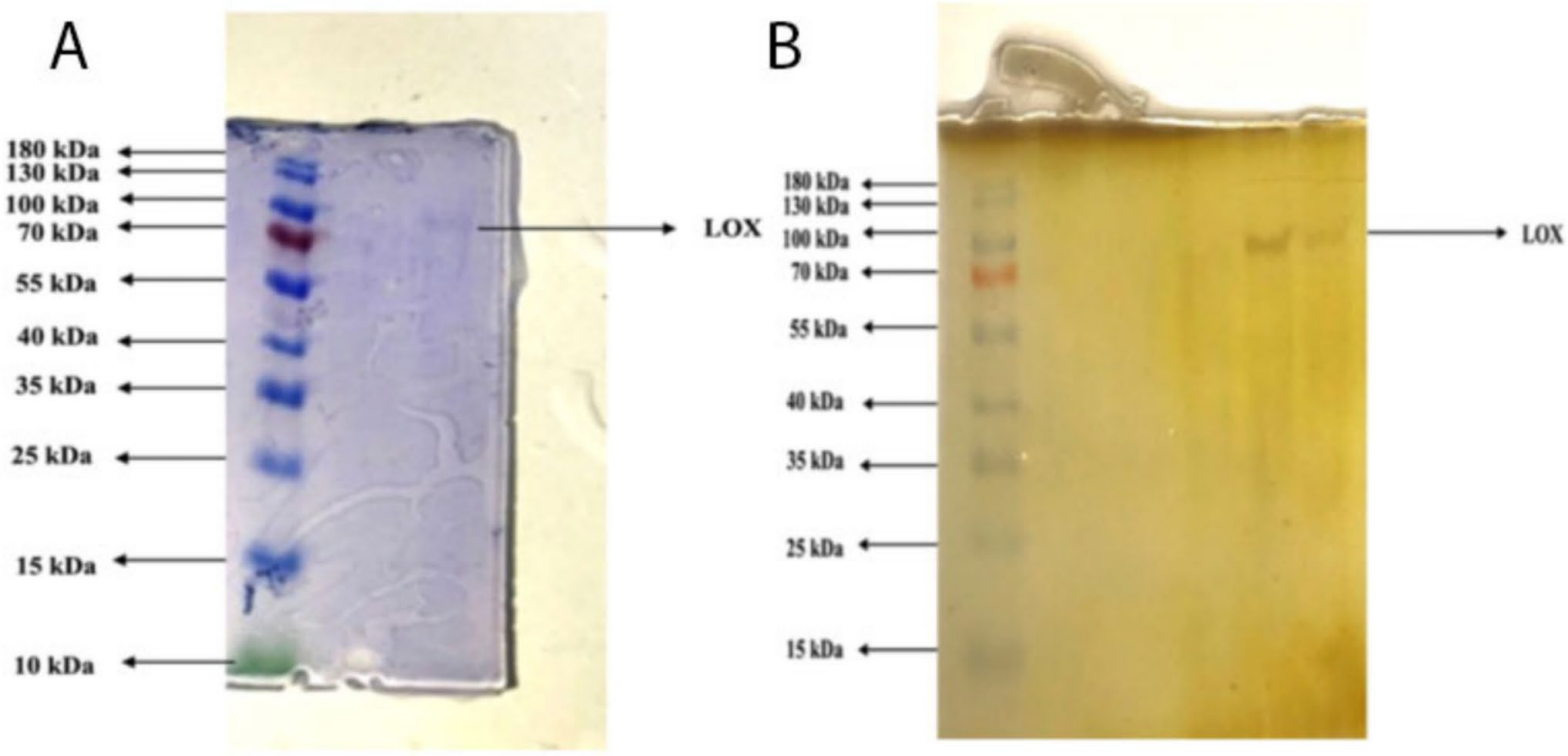
SDS-PAGE image for LOX enzyme A: obtained by Coomassie blue staining method B: obtained by silver staining method

This work included the purification of the LOX enzyme via three stages: homogenate preparation, ammonium sulfate precipitation, and Q-sepharose anion exchange chromatography. To determine the optimal ammonium sulfate fractionation range for LOX precipitation, stepwise saturations were performed at 20%, 40%, 60%, and 80% levels (Kirici et al. 2016). Enzymatic activity assays revealed that the 20–40% saturation range provided the highest specific LOX activity and protein enrichment. Therefore, this interval was selected for the subsequent purification steps to ensure optimal recovery of functional enzyme. Table 3 indicates that the LOX enzyme recovered from quinoa had a specific activity of 1.48, a yield of 7.24%, and a purification fold of 77.89. The molecular weight of the isolated LOX enzyme was determined to be around 97 kDa (Fig. 1). The optimum pH for LOX enzyme in homogenate obtained from quinoa seeds was determined as 5.5. The optimum ionic strength for LOX enzyme obtained from quinoa seeds was determined as 0.9 M.

**Table 3:** Purification steps of LOX enzyme from quinoa

Purification step	Activity (EÜ/mL)	Protein (mg/mL)	Total Volume (mL)	Total Activity (EU)	Total Protein (mg)	Specific Activity (EÜ/mg protein)	Purification Fold	% Yield
Homogenate	0.184	9.58	25	4.60	239.50	0.019	1	100
(NH_4_)_2_SO_4_ and Dialysis	0.120	4.13	15	1.80	61.95	0.029	1.53	39.13
Q-sepharose ion exchange chromatography	0.037	0.025	9	0.33	0.225	1.48	77.89	7.24

The literature contains research on the purification of LOX enzyme from many sources and investigations into the inhibitory effects of certain chemicals on its activity. LOX has been isolated from durum wheat a yield of 68.2% (Barone et al. 1990) and eggplant (López-Nicolás et al. 2001). In a study of pea (*Pisum sativum* L., cv. Alaska) mitochondria, the LOX enzyme was isolated using SDS-PAGE, exhibiting a molecular mass of 97 kDa (Braidot et al. 2004). Pérez-Gilabert et al. (Pérez-Gilabert et al. 2005) isolated the LOX enzyme from the desert truffle (*Terfezia claveryi*), a mycorrhizal fungus that mitigates lipid rancidity and prolongs storage life due to its elevated polyunsaturated fatty acid (PUFA) content, utilizing phase separation followed by two-stage cation exchange chromatography in the presence of Triton X-114. The enzyme's molecular mass was ascertained to be 66 kDa via SDS-PAGE; they noted its specific affinity for linoleic and linolenic acid, demonstrating 32% activity with linoleic acid. LOX enzyme recovered from desert truffle had a purification fold of 52.1. In another study, the LOX enzyme, having a molecular mass of 93 kDa, was isolated from pea seeds (*Pisum sativum* var. *Telephone* L.) using SDS-PAGE, employing DEAE-Sephadex A-50 ion exchange chromatography and ammonium sulfate precipitation (0–80% saturation). LOX enzyme recovered from pea seeds had a yield of 22.29% and a purification fold of 47.16 (Szymanowska et al. 2009a). Another work included the partial purification and characterization of the LOX enzyme from avocado (*Persea americana* Mill. cv. Hass) using ion exchange chromatography with a purification fold of 3.4 (Jacobo-Velázquez et al. 2010).

### LOX inhibition by the synthesized Schiff base ligands and their metal complexes

**Fig. 2:**
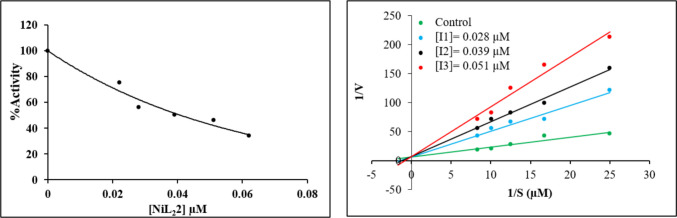
IC_50_ and Lineweaver–Burk graphs of the best inhibitor for LOX

In this article, the effects of synthesized schiff bases on purified LOX enzyme were also investigated. In our study, two different ligands as L^1^H and L^2^H were used, and their metal complexes were synthesized. In the metal complexes of L^1^H ligand, no inhibition effect was observed for other metals except nickel and cadmium. All the compounds studied exhibited competitive inhibition. The results indicated that Ni(L^2^)_2_ exhibited the highest inhibitory activity against the LOX enzyme among the test compounds (Table 4, Fig. 2).

**Table 4:** IC_50_ values and K_i_ constants of newly synthesized schiff base and metal complex acting against LOX enzyme activity

Inhibitor	IC_50_ (μM)	R^2^	*K*_i_ (μM)	Inhibition Type
L^1^H	0.930 ± 0.271	0.9826	0.858 ± 0.194	Competitive
CdL^1^	0.502 ± 0.105	0.9798	0.519 ± 0.014	Competitive
NiL^1^	0.253 ± 0.098	0.9670	0.448 ± 0.087	Competitive
L^2^H	0.317 ± 0.032	0.9662	0.024 ± 0.003	Competitive
NiL^2^	0.257 ± 0.054	0.9850	0.014 ± 0.002	Competitive
CoL^2^	0.071 ± 0.006	0.9682	0.068 ± 0.014	Competitive
CuL^2^	0.064 ± 0.008	0.9676	0.091 ± 0.042	Competitive
ZnL^2^	0.058 ± 0.01	0.9834	0.067 ± 0.017	Competitive
UO_2_L^2^	0.032 ± 0.003	0.9873	0.047 ± 0.011	Competitive
CdL^2^	0.043 ± 0.007	0.9728	0.043 ± 0.008	Competitive
Caffeic acid*	8.21 ± 0.98	0.9678	12.67 ± 1.44	Noncompetitive

*Standard inhibitor for LOX

In two different ligands, nickel complex inhibited LOX enzyme more than other metals. The superior inhibitory activity of the Ni(L^2^)_2_ complex may be attributed to several structural factors. Firstly, the square planar geometry commonly adopted by Ni(II) Schiff base complexes ensures optimal planarity and electron delocalization, which can enhance binding affinity toward the LOX active site. Additionally, the L^2^H possesses extended π-conjugation and electron-donating substituents that may increase the metal–ligand stability and favor interactions with key amino acid residues as shown in the molecular docking simulations. Compared to other metal complexes, Ni(L^2^)_2_ demonstrated lower binding energy and more hydrogen bonding interactions with catalytically relevant residues, supporting its high potency. These structural and electronic features likely contribute to the complex's strong inhibitory activity observed in both in vitro and in silico analyses. Coordination of L^2^H to Ni(II) plays a decisive role in enhancing inhibitory activity. Ni(II) complexes typically adopt geometries that favor planarity and π-electron delocalization, which can facilitate optimal stacking and π–cation interactions with catalytically relevant residues such as Lys278. In the present case, Ni(L^2^)₂ establishes a denser interaction network than its L^1^H counterpart, including multiple hydrogen bonds involving Val256, Arg260, and Thr274, in addition to conserved hydrophobic contacts within the LOX binding cavity.The use of zinc and copper complex did not show much change in enzyme inhibition. The cadmium L^2^H complex exhibited about 2 times more inhibition effect than the copper complex (Table 4).

There are studies on the inhibition of LOX enzyme in the literature. Öztürk Kesebir (Öztürk Kesebir 2022) demonstrated that vanillic acid, syringaldehyde, p-coumaric acid, and morin hydrate, inhibited LOX, with IC_50_ values of 218.1 μM, 643.2 μM, 233 μM, and 304 μM, respectively. Yashaswini et al. (Yashaswini et al. 2017) discovered that sesamol inhibited the soybean LOX-1 enzyme in a dose-dependent manner, with an IC_50_ value of 51.84 μM and a K_i_ of 4.9 μM.

Khan et al. (Khan et al. 2015) examined the inhibitory effects of the aerial components of *Polygonatum verticillatum* on acetylcholinesterase, urease, butyrylcholinesterase, and LOX enzymes using standardized experimental methodologies. The investigation revealed that the extracts had a substantial impact against the LOX enzyme. Fazal-Ur-Rehman et al. (Fazal-ur-Rehman et al. 2019) produced a variety of oxamide ligands by the reaction of amines with oxalyl chloride in a basic media. Spectroscopic and analytical methods, including IR, ^1^H-NMR, and ESI–MS, were used for the characterisation of the produced oxamides. The produced oxamides were evaluated for their inhibitory effects on the LOX enzyme. Biological screening demonstrated that the oxamides exhibited substantial LOX inhibition, but the unsubstituted oxamides shown no significant LOX inhibition. Abdelkhalek et al. (Abdelkhalek et al. 2023) designed and synthesized a new series of thieno[2,3-d]pyrimidine derivatives starting from cyclohexanone under Gewald condition to develop multi-targeted ligands against both 15-LOX and COX-2 enzymes with anti-inflammatory properties. Compound **6o** exhibited more potent 15-LOX inhibition (IC_50_ = 1.17 μM) than the reference nordihydroguaiaretic acid (NDGA, IC_50_ = 1.28 μM). Furthermore, molecular docking studies for compounds binding to the active sites of 15-LOX and COX-2 showed good agreement with biological evaluations. Moreover, Alavi et al. (Alavi et al. 2021) designed, synthesized and evaluated a series of monovalent diallylphenol derivatives as potential 15-hLOX-1 inhibitors. The electronic natures of the allyl moiety and para substituents were found to play a major role in the radical scavenging activity and subsequent LOX inhibition potential of the synthetic inhibitors. Among the synthetic compounds, 2,6-diallyl-4-(hexyloxy)phenol (IC_50_ = 0.88 μM) and 2,6-diallyl-4-aminophenol (IC_50_ = 0.80 μM) showed the best results for LOX inhibition. Another study, Kaempferol, quercetin, benzoic acid, ferulic acid, catechin, and caffeic acid, were shown to inhibit the LOX enzyme in the following order of efficacy: caffeic acid > quercetin > catechin > benzoic acid > ferulic acid > kaempferol (Szymanowska et al. 2009b).

### In silico* studies*

Molecular docking enables the identification of plausible binding modes of ligands within protein cavities and provides residue-level interaction hypotheses that may help rationalize structure–activity trends. In this study, docking simulations were performed to interpret the binding of selected inhibitors to the LOX active site and to relate the predicted interaction patterns to the in vitro kinetic inhibition data. The docking protocol was validated by re-docking the co-crystallized protocatechuic acid; the RMSD between the crystallographic pose and the re-docked pose was 0.18 Å, supporting reliable pose retrieval within the selected grid.

Docking was carried out using the Glide XP algorithm, and docking scores are reported as GlideScore values (kcal/mol), where more negative values indicate a more favorable predicted fit within the defined binding region. To complement docking-based ranking, top poses were rescored using Prime MM-GBSA to estimate ΔG_bind_. It should be noted that docking/MM-GBSA scores are approximations and may not fully capture entropic effects, explicit-solvent contributions, and metal-associated interaction terms; therefore, the results were interpreted primarily at the level of binding mode and residue-level contacts and discussed alongside the experimental kinetic parameters.

Although multiple metal complexes showed notable LOX inhibition, Ni(L^2^)_2_ and Ni(L^1^)_2_ were selected for detailed docking and DFT analyses due to their representative structural and electronic features and because both share comparable bidentate chelation and similar octahedral coordination environments. This selection allowed a controlled comparison of how ligand substitution patterns (L^1^H vs L^2^H) may modulate LOX binding while minimizing variability arising from different metal identities, coordination geometries, or oxidation states. Experimentally, Ni(L^2^)2 was the most potent inhibitor (K_i_ = 0.014 ± 0.002 µM), whereas Ni(L^1^)2 showed moderate inhibition (K_i_ = 0.448 ± 0.087 µM).

**Fig. 3:**
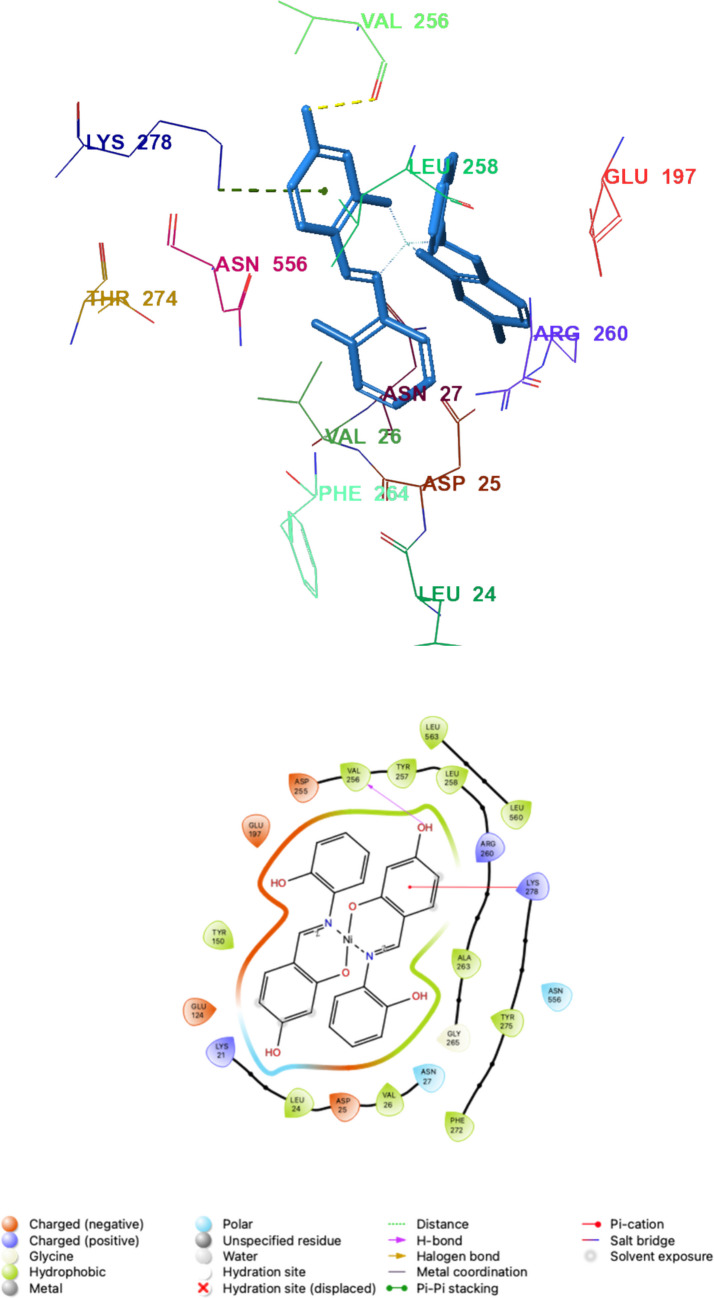
3D and 2D interaction diagrams of Ni(L^1^)_2_—LOX (PDB ID: 1N8Q) obtained as a result of molecular docking (Hydrogen bond is shown in yellow and π-cation interaction is shown in green dashed lines)

**Fig. 4:**
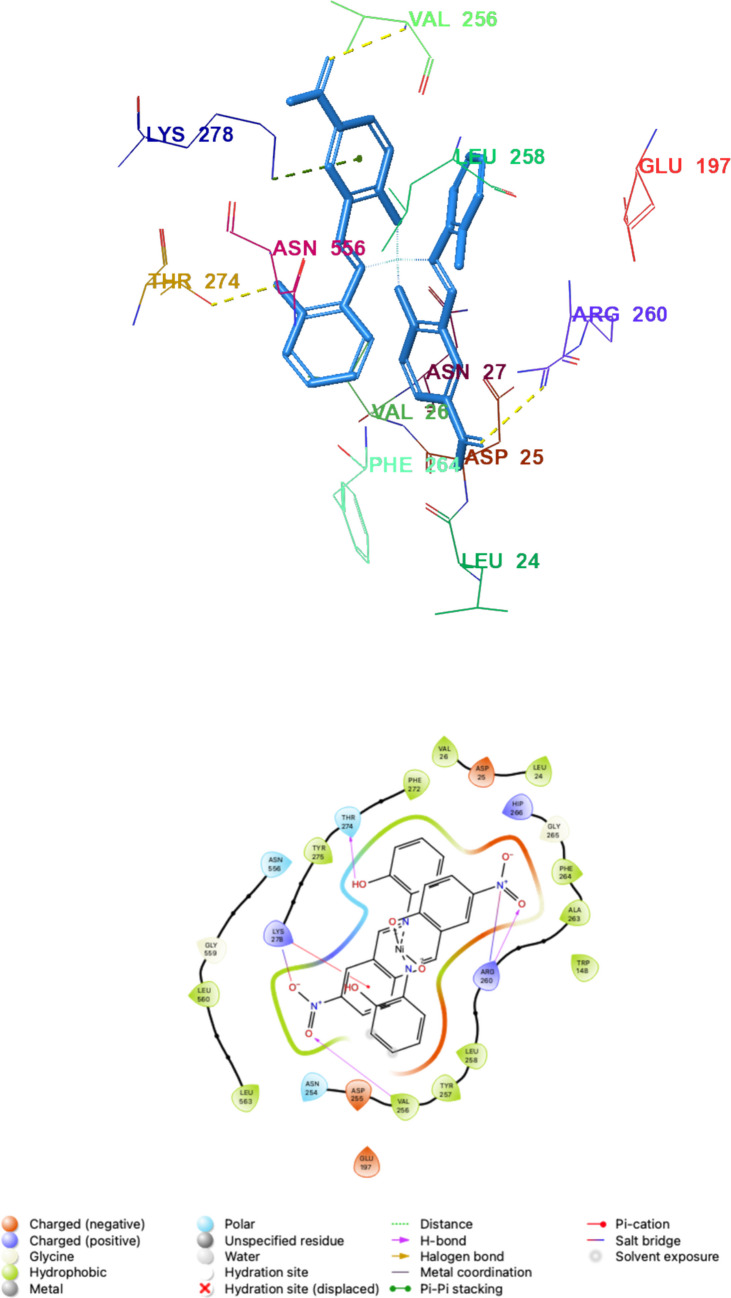
3D and 2D interaction diagrams of Ni(L^2^)_2_—LOX (PDB ID: 1N8Q) obtained as a result of molecular docking (Hydrogen bond is shown in yellow and π-cation interaction is shown in green dashed lines)

In the binding cavity, Ni(L^1^)2 was stabilized by a hydrogen bond between the hydroxyl group and Val256 (2.00 Å) and a π–cation interaction involving the aromatic system and the catalytically relevant Lys278, supported by extensive hydrophobic contacts (Leu24, Val26, Trp148, Tyr150, Val256, Tyr257, Leu258, Ala263, Phe264, Phe272, Tyr275, Leu560, and Leu563). In contrast, Ni(L^2^)2 established a denser polar interaction network: hydrogen bonds formed between the nitro group(s) and Val256 (2.07 Å) and Arg260 (2.09 Å), along with an additional hydrogen bond between the hydroxyl group and Thr274 (1.82 Å). Similar to Ni(L^1^)2, Ni(L^2^)2 also displayed a π–cation interaction with Lys278 and comparable hydrophobic packing within the same residue cluster, indicating that both complexes adopt similar cavity occupancy while differing in their polar anchoring pattern (Figs. 3 and 4).

These residue-level interactions are consistent with the experimental inhibition trend: the additional hydrogen-bonding contacts observed for Ni(L^2^)2 (Arg260 and Thr274) provide a plausible structural rationale for its markedly stronger competitive inhibition relative to Ni(L^1^)2.

Among the two Ni(II) complexes, Ni(L^1^)2 exhibited a more favorable docking score (–6.38 kcal/mol) and MM-GBSA binding energy (–12.30 kcal/mol) than Ni(L^2^)2 (–4.54 and –9.88 kcal/mol, respectively). Despite this, Ni(L^2^)2 showed substantially stronger experimental inhibition (Ki = 0.014 µM vs 0.448 µM), suggesting that static scoring may not fully represent the determinants of potency for these metal-containing systems. Differences in solvation/desolvation, conformational flexibility, and metal-associated interaction components may contribute to the experimental activity beyond what is captured by rigid docking/scoring. Accordingly, docking is used here primarily to rationalize binding poses and key contacts, whereas the kinetic data provide the definitive potency ranking.

The selection of PDB ID: 1N8Q was based on structural and functional considerations. This structure provides a high-resolution crystallographic model (2.1 Å) co-crystallized with protocatechuic acid, which delineates the inhibitor-binding region and enables robust grid definition and RMSD-based docking validation. Additionally, the structure contains the catalytically relevant non-heme iron center coordinated by conserved residues, supporting a realistic representation of the active-site architecture. While other LOX-related structures exist in the PDB, they may lack bound inhibitors, have lower resolution, or represent proteins with different active-site geometries; therefore, 1N8Q was deemed an appropriate template for reproducible docking in a functionally annotated LOX binding pocket.

**Fig. 5:**
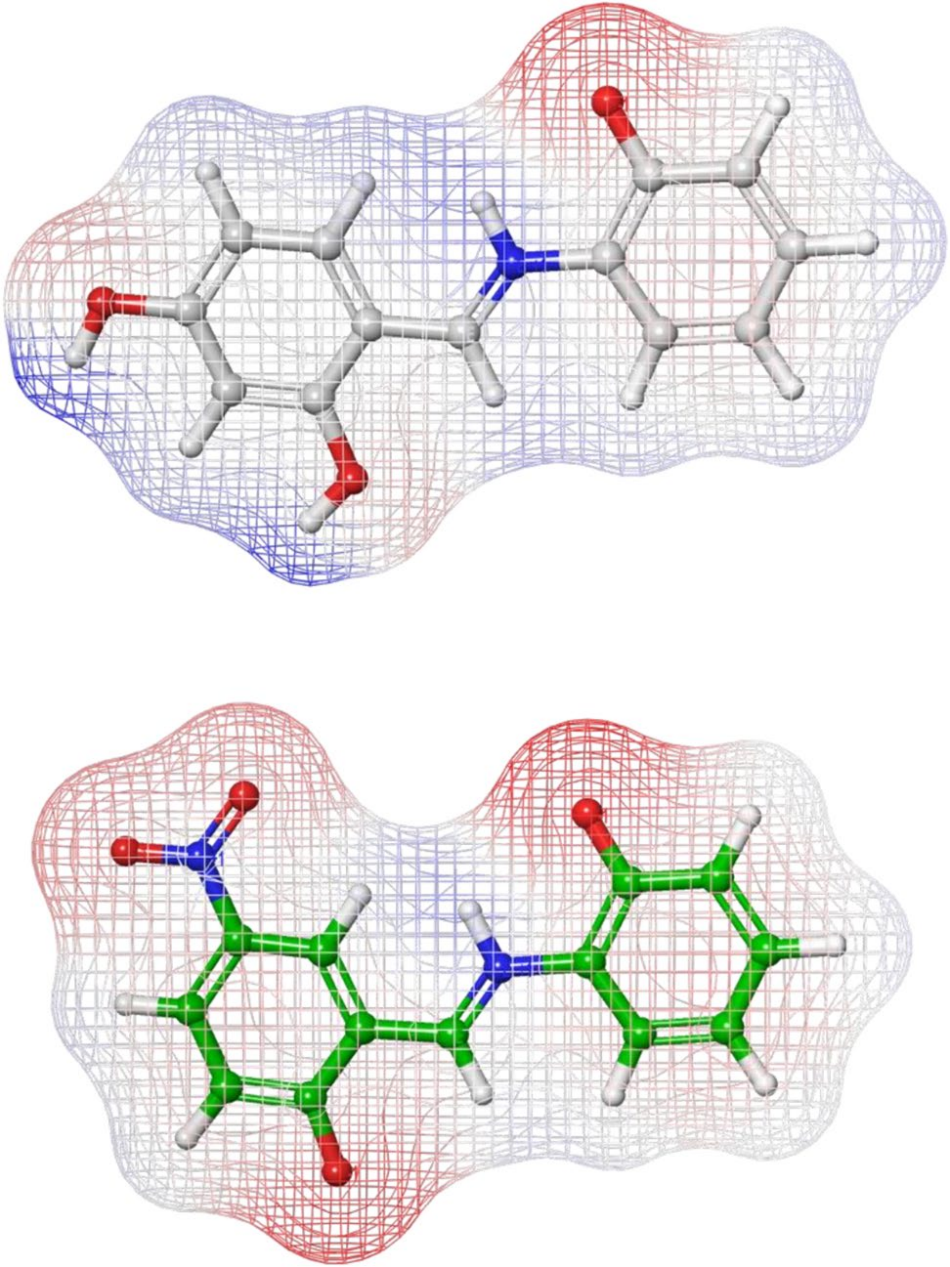
Electrostatic potential (ESP) maps of L^1^H (top) and L^2^H (bottom). The spatial topology was computed via Jaguar at the B3LYP/6-311G +  + ** level of theory

**Fig. 6:**
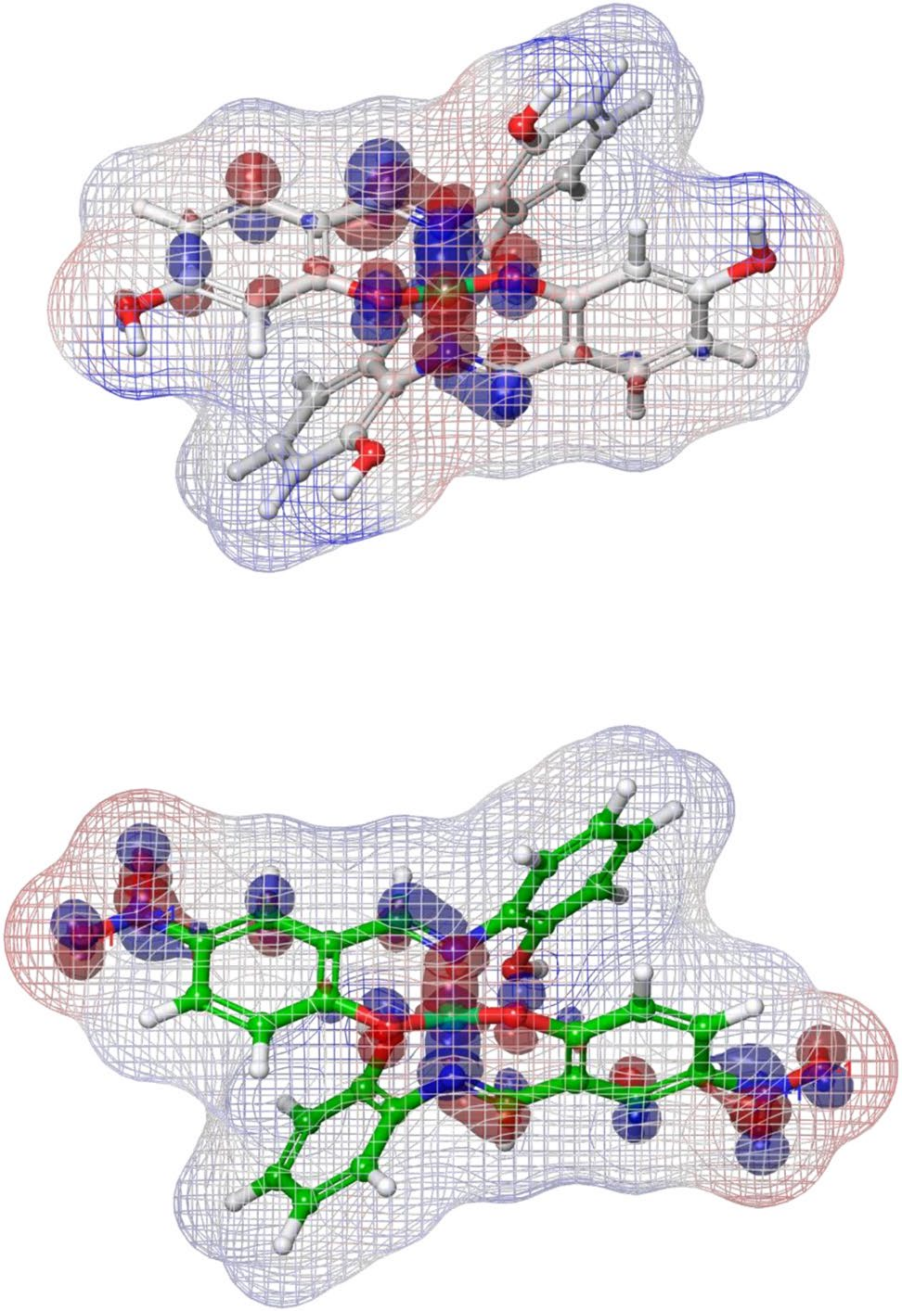
Combined frontier molecular orbital and electrostatic potential (ESP) maps of Ni(L^1^)_2_ (top) and Ni(L^2^)_2_ (bottom). The spatial topology was computed via Jaguar at the B3LYP/3-21G* level of theory

To further probe electronic features potentially relevant to binding and reactivity, Ni(L^1^)2, Ni(L^2^)2 and their parent ligands (L^1^H and L^2^H) were evaluated by DFT calculations using the Jaguar module at the B3LYP/3-21G* and B3LYP/6-311G +  + ** levels of theory, respectively. The computed HOMO and LUMO energies were − 0.119275 and − 0.049315 eV for Ni(L^1^)2, and − 0.139491 and − 0.091099 eV for Ni(L^2^)2, indicating a narrower HOMO–LUMO gap for Ni(L^2^)2. As shown in Fig. 5, ESP maps of L^1^H and L^2^H revealed localized negative potential regions around the phenolic oxygen and nitro substituents, consistent with their involvement in hydrogen bonding and electrostatic contacts within the binding cavity. Figure 6 overlays frontier orbital densities onto ESP surfaces of the Ni(II) complexes and supports the interaction-oriented interpretation derived from docking, in agreement with the in vitro inhibition profile.

## Conclusion

This study evaluated the LOX inhibitory potential of a series of Schiff base ligands derived from salicylaldehyde and their corresponding metal complexes. The purified quinoa LOX enzyme (~ 97 kDa) enabled a consistent biological platform for in vitro assays. Among the synthesized compounds, metal complexes exhibited notably higher inhibitory activities than their corresponding ligands, suggesting a synergistic effect between the metal center and the azomethine moiety. Molecular docking studies further supported these findings by indicating stable coordination between the metal centers and key residues in the LOX active site, reinforcing the proposed mechanism of inhibition. These results suggest that salicylaldehyde-derived Schiff base metal complexes are promising scaffolds for further development of LOX inhibitors. Beyond the specific compounds reported here, the findings of this study provide a generalizable framework for the rational design of metal-assisted enzyme inhibitors. This approach may inform future efforts aimed at developing more potent and selective LOX inhibitors, exploring structure–activity relationships across different LOX isoforms, and extending metal–Schiff base strategies to other therapeutically relevant metalloenzymes. Consequently, the present work contributes not only new inhibitory candidates but also conceptual insights that may guide future research in enzyme-targeted drug discovery. Future studies may focus on expanding the ligand library, evaluating selectivity against human LOX isoforms, and validating biological activity through in vivo and cell-based models.

## Experimental

### Chemistry

2,4-dihydroxybenzaldehyde (98%, Merck), 2-hydroxy-5-nitrobenzaldehyde (98%, Merck), o-aminophenol (99%, Merck), cobalt(II) acetate tetrahydrate (99%, Merck), nickel(II) acetate tetrahydrate (99.9%, Sigma-Aldrich), copper(II) acetate monohydrate (99%, Merck), zinc(II) acetate dihydrate (99.5%, Merck), cadmium(II) acetate dihydrate (98%, Merck), uranyl acetate (VI) dihydrate (98%, Merck), p-toluenesulfonic acid (98%, Merck) and solvents (98–99.9%, Merck) were used for synthesis of ligands and metal complexes.

The IR spectra of the synthesized compounds were recorded using Thermo Scientific Nicolet 6700 FT-IR Spectrophotometer in the range of 4000–400 cm^−1^ by using pellets prepared with KBr in solid form. Each spectrum was collected by averaging 32 scans with a resolution of 4 cm^−1^**,** using a DTGS (Deuterated Triglycine Sulfate) detector under ambient conditions.

Elemental analyzes were taken at LECO-932 CHNSO model elemental analyzer. The ^1^H-NMR and ^13^C-NMR spectra of all synthesized Schiff base ligands and their metal complexes were recorded in DMSO-d₆ on a Bruker DPX-400 (400 MHz) spectrometer. UV–Vis absorption spectra were measured in ethanol for free ligands (L^1^H and L^2^H) and in DMSO for their corresponding metal complexes, using a Shimadzu UV-1800 spectrophotometer in the range of 200–800 nm. The electronic studies were taken with DMSO and EtOH and recorded on a Shimadzu 1240 model UV–Vis Spectrophotometer. Magnetic susceptibilities were measured at room temperature by a modified Gouy method by using Hg[Co(SCN)_4_] for calibration. In addition, FEI QUANTA FEG 450 model Scanning Electron Microscope (SEM) was used to visualize the structure and the Panalytical Empyrean X-Ray Diffraction Device (XRD) was used for X-ray diffraction data.

### Sample preparation for characterization

The Schiff base ligands and their metal complexes were dried in vacuo over phosphorus pentoxide (P_2_O_5_) at room temperature for 24 h prior to elemental (C, H, N) analysis to eliminate moisture and solvent residues. IR spectra were recorded using KBr pellet method for ligands, while metal complexes were analyzed either as KBr pellets or using ATR mode, depending on solubility and sample homogeneity. Ligands were dissolved in deuterated DMSO (DMSO-d_6_) at a concentration of ~ 10 mg/mL. Samples were filtered through 0.22 µm PTFE filters prior to NMR measurements (for ligands) to remove any particulate matter.

### Synthesis of ligands

#### ***(E)−4-(((2-hydroxyphenyl)imino)methyl)benzene-1,3-diol [L***.^***1***^***H]***

**Scheme 1:**
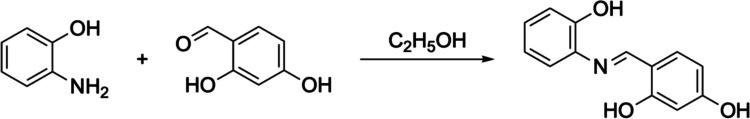
Synthesis of Schiff base L^1^H, (E)−4-(((2-hydroxyphenyl)imino)methyl)benzene-1,3-diol

The Schiff base ligand was prepared by condensing 2,4-dihydroxybenzaldehyde (1.38 g, 10 mmol) and *o*-aminophenol (1.09 g, 10 mmol). 20 mL ethanolic solution of 2,4-dihydroxybenzaldehyde was slowly added to 20 mL ethanolic solution of *o*-aminophenol and *p*-toluenesulfonic acid (Maxim et al. 2008; Devi et al. 2019). Then mixture was refluxed for 3 h. The carbonyl peak of the aldehyde was followed by IR spectrometer. The reaction progress was monitored by FT-IR by tracking the disappearance of the aldehyde C = O stretching band (~ 1680–1700 cm⁻^1^) and the concomitant appearance/intensification of the azomethine (C = N) band (~ 1612 cm⁻^1^). Completion was further confirmed by TLC (silica gel, UV detection), where the aldehyde spot disappeared. The precipitate obtained was filtered, washed with cold water and ether. And then, recrystallization from ethanol afforded pure Schiff base. The synthesis of Schiff base L^1^H is shown in Scheme 1.

FT**-**IR (KBr disk, *ν* cm^−1^); 3430 (O–H), 3056 (C-H, aromatic), 2942–2910 (C-H, aliphatic), 1612 (C = N), 1528–1420 (C = C, aromatic), 1272 (C-O) and 1090 (C-O). ^1^H NMR (DMSO-d_6_); δ 12.89 (s, 1H), 9.66 (s, 1H), 8.84 (s, 1H), 7.34 (s, 1H), 7.12 (d, 1H), 7.00 (d, 1H), 6.96 (d, 1H), 6.87 (d, 1H), 6.76 (d, 1H). ^13^C NMR (DMSO-d_6_); δ 162.0, 153.8, 151.5, 149.8, 135.9, 128.3, 121.1, 120.1, 120.0, 119.9, 117.6, 117.3, 116.9

#### ***(E)−2-(((2-hydroxyphenyl)imino)methyl)−4-nitrophenol [L***.^***2***^***H]***

**Scheme 2:**
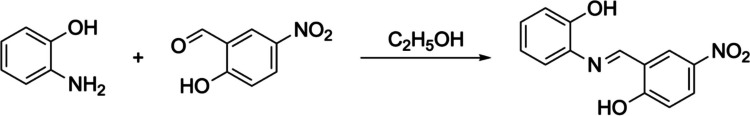
Synthesis of Schiff base L^2^H, (E)−2-(((2-hydroxyphenyl)imino)methyl)−4-nitrophenol

The Schiff base ligand was prepared by condensing 2-hydroxy-5-nitrobenzaldehyde (1.67 g, 10 mmol) and *o*-aminophenol (1.09 g, 10 mmol). 20 mL ethanolic solution of 2-hydroxy-5-nitrobenzaldehyde was slowly added to 20 mL ethanolic solution of *o*-aminophenol and *p*-toluenesulfonic acid (Maxim et al. 2008; Devi et al. 2019). Then mixture was refluxed for 4 h. The carbonyl peak of the aldehyde was followed by IR spectrometer. Reaction completion was confirmed by FT-IR by the disappearance of the aldehyde C = O band (~ 1680–1700 cm⁻^1^) and the appearance of the characteristic azomethine (C = N) band (~ 1630 cm⁻^1^). TLC (silica gel, UV detection) also indicated completion by the absence of the starting aldehyde spot. The precipitate obtained was filtered, washed with cold water and ether. And then, recrystallization from ethanol afforded pure Schiff base. The synthesis of Schiff base L^2^H is shown in Scheme 2.

FT**-**IR (KBr disk, ν cm^−1^); 3435 (O–H), 3074 (C-H, aromatic), 2940–2910 (C-H, aliphatic), 1630 (C = N), 1528–1420 (C = C, aromatic), 1294 (C-O) and 1092 (C-O). ^1^H NMR (DMSO-d_6_); δ 15.74 (s, 1H), 10.39 (s, 1H), 9.31 (s, 1H), 8.62 (d, 1H), 8.38 (m, 1H), 8.17 (d, 1H), 7.59 (d, 1H), 7.19 (m, 1H), 6.68 (m, 1H). ^13^C NMR (DMSO-d_6_); δ 164.0, 159.1, 151.3, 148.89, 141.3, 131.4, 129.5, 120.2, 120.2, 119.5, 117.1, 91.7, 78.9

### Synthesis of metal complexes

**Scheme 3:**
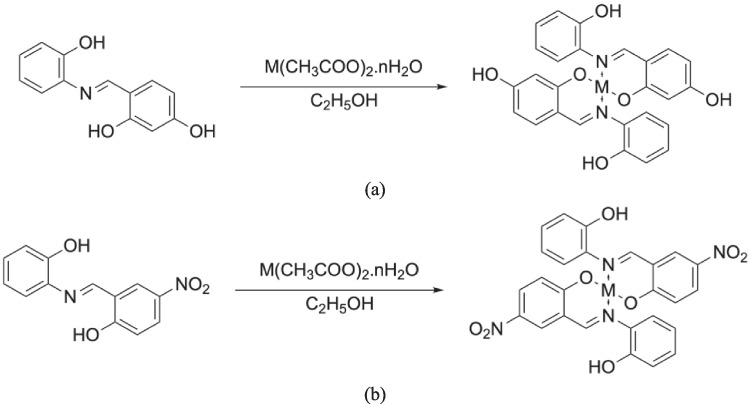
Synthesis of metal complexes of Schiff Base L^1^H (**a**) and L.^2^H (**b**) [M: Co(II), Ni(II), Cu(II), Zn(II), Cd(II), UO_2_(VI)]

The metal complexes were synthesized by refluxation-precipitation method, in which the metal salt and the Schiff base ligand were heated under reflux to promote complex formation, followed by gradual precipitation of the product upon cooling, allowing for efficient isolation and purification of the complexes. The hot ethanolic solution of Schiff base ligand (0.002 mol) was mixed with ethanolic solution of metal acetate [cobalt(II) acetate tetrahydrate (0.2490 g, 0.001 mol), nickel(II) acetate tetrahydrate (0.2488 g, 0.001 mol), copper(II) acetate monohydrate (0.1996 g, 0.001 mol), zinc(II) acetate dihydrate (0.2195 g, 0.001 mol), cadmium (II) acetate dihydrate (0.2665 g, 0.001 mol), uranyl (VI) acetate dihydrate (0.4261 g, 0.001 mol)]. The reaction mixture was refluxed for 4–6 h. The respective metal complexes were filtered, washed with water, then with 5% ethanol and were dried in vacuum over anhydrous. The synthesis of Schiff base complexes is shown in Scheme 3.

### Homogenization of the quinoa (Chenopodium qunioa Willd.) plant

Twenty grams of quinoa sourced from Erzurum herbalists were milled into flour using a grinder, and eighty milliliters of a 0.2 M phosphate buffer solution (pH 7) at a 1:4 weight/volume ratio, comprising 5% NaCl and 1% acetone, was maintained at 40 °C for sixty minutes. The quinoa was subjected to homogenization for 5 min using ultrasound. Subsequently, samples were transferred into centrifuge tubes and subjected to centrifugation at 13.000 g for 30 min, after which the supernatant was collected.

### Assessment of LOX enzyme activity

The techniques established by Arens et al. (Arens et al. 1973) and Barone et al. (Barone et al. 1990) were used to assess LOX activity. The approach included the preparation of a linoleic acid substrate solution in 5 mL of methanol at a concentration of 4 µM. 2910 μL (90 μL of substrate solution was combined with 50 mM phosphate buffer (pH 6.5), followed by the addition of 20 μL of enzyme solution, and the absorbance at 234 nm was measured for a duration of 3 min.

### Purification of LOX enzyme

LOX enzyme was partially purified from *Chenopodium quinoa* seeds using a three-step protocol including homogenization, ammonium sulfate fractionation (20–40% saturation), and anion exchange chromatography on a Q-Sepharose column. Anion exchange chromatography was performed on a Q-Sepharose column (1.0 × 10 cm) pre-equilibrated with 50 mM phosphate buffer (pH 7.0). Protein samples were loaded at a flow rate of 0.5 mL min⁻^1^, and bound proteins were eluted using a linear NaCl gradient (0–1.0 M) at the same flow rate. Fractions of 1.0 mL were collected and assayed for LOX activity. Each purification step was followed by enzymatic activity assays and protein quantification. The eluates were monitored spectrophotometrically, and active fractions were pooled for subsequent analyses. Further experimental details are provided in the Supplementary Material.

### *Determination of IC*_*50*_* and K*_*i*_

The IC_50_ values were determined by measuring the residual enzyme activity at five different inhibitor concentrations. The percentage of remaining LOX activity was plotted against the logarithm of inhibitor concentration, and the IC₅₀ values were calculated by fitting the data to a dose–response curve using nonlinear regression in GraphPad Prism software (version 9.0). Kᵢ values were calculated from Lineweaver–Burk plots using nonlinear regression analysis implemented in GraphPad Prism (version 9.0) (Lineweaver and Burk 1934). All enzymatic measurements were performed in triplicate (n = 3), and results are expressed as mean ± standard deviation.

### In silico study

The three-dimensional structure of the LOX enzyme (PDB ID: 1N8Q) (Borbulevych et al. 2004) was obtained from the “RCSB Protein Data Bank” website (https://www.rcsb.org) and prepared using the Protein Preparation Wizard module of the Small-Molecule Drug Discovery Suite (Schrödinger, LLC, NY, USA) software (Madhavi Sastry et al. 2013). The protocatechuic acid, which was crystallized together with the enzyme, was retained at the enzyme’s binding site. Missing hydrogen atoms in the structure were added using the Prime module (Jacobson et al. 2004) of the same software, the ionization and tautomeric states of amino acid residues were determined using the Epik module (Shelley et al. 2007), and the proton orientations were adjusted at a pH of 7.0 ± 2.0 using the PROPKA module. During the protein preparation process, only the water molecules at the active site of the enzyme were retained, and those located more than 5 Å from the ligand were removed. The positions of these water molecules in the binding region were then re-optimized using the OPLS4 force field (Lu et al. 2021). The two-dimensional structures of the synthesized compounds were drawn using ChemDraw 21 (PerkinElmer, Inc., Waltham, MA, USA) software, and their possible ionization states, tautomers, and enantiomers at pH 7.0 ± 2.0 were determined with the help of the LigPrep module (Sharanya et al. 2018). The minimization of ligands was performed using the OPLS4 force field parameters. Prior to docking, the binding region map was created using the Receptor Grid Generation module (Halgren 2009) and docking was performed using the Glide XP algorithm (Friesner et al. 2004; Türkeş 2024, 2026). Docking scores are reported as GlideScore values (kcal/mol), and Prime MM-GBSA was used for post-docking rescoring to estimate ΔG_bind_ for the selected poses. Additionally, quantum chemical calculations were carried out using Schrödinger Jaguar to investigate the electronic properties and chemical reactivity of L^1^H_,_ L^2^H, Ni(L^1^)_2_, and Ni(L^2^)_2_. Geometry optimization was performed in the gas phase using the hybrid B3LYP functional with the 6-311G +  + **/3-21G* basis sets.

## Supplementary Information

Below is the link to the electronic supplementary material.

Supplementary file1 (DOCX 1005 KB)

## Data Availability

Data will be made available on request.
