# Supplementary material for: Synthesis, characterization, and lipoxygenase inhibition of salicylaldehyde-derived schiff base metal complexes: enzymatic and in silico evaluation using quinoa lipoxygenase
Source: Naunyn Schmiedebergs Arch Pharmacol. 2026 Jan 16;399(6):8975–90. doi: 10.1007/s00210-025-04955-6 (PMC13086667; doi:10.1007/s00210-025-04955-6)
Supplement: Supplementary file 1 — Supplementary file1 (DOCX 1005 KB) [file 210_2025_4955_MOESM1_ESM.docx]

**Synthesis, Characterization, and Lipoxygenase Inhibition of Salicylaldehyde-Derived Schiff Base Metal Complexes: Enzymatic and In Silico Evaluation Using Quinoa Lipoxygenase**

Ergün Ereminsoy ^a^, Yeliz Demir ^b,*^, Sümeyra Tuna Yıldırım ^c,^, Cüneyt Türkeş ^d^, Ömer İrfan Küfrevioğlu ^a,*^

^a^ Department of Chemistry, Faculty of Science, Atatürk University, Erzurum 25240, Türkiye

^b^ Department of Pharmacy Services, Nihat Delibalta Göle Vocational High School, Ardahan University, Ardahan 75700, Türkiye

^c^ Department of Analytical Chemistry, Faculty of Pharmacy, Erzincan Binali Yıldırım University, Erzincan 24002, Türkiye

^d^ Department of Biochemistry, Faculty of Pharmacy, Erzincan Binali Yıldırım University, Erzincan 24002, Türkiye

Purification of LOX enzyme

The homogenate obtained from solid ammonium sulfate and quinoa was precipitated at 20-unit intervals starting from 20% to 80%, respectively. The precipitation mixture was centrifuged at +4°C, 13000g for 15 minutes. The pellets were dissolved in homogenate buffer and the precipitation range was determined by activity measurement. The column filler material used in Q-Sepharose anion exchange chromatography was easily accessible. The column was equilibrated with a 50 mM sodium phosphate buffer at pH 6.5. The sample acquired post-ammonium sulfate precipitation was introduced to the column, which was then washed with the same buffer. Eluates exhibiting elevated LOX activity were obtained using incremental NaCl elution. The protein concentrations in the enzyme solution, purified using homogenization, ammonium sulfate precipitation, and Q-Sepharose anion exchange chromatography from the quinoa plant, were quantified using the Bradford technique. The purity of LOX enzyme purified by SDS-PAGE was checked.

**Determination of optimum pH and ionic strength**

For linoleic acid substrate, the optimum pH for LOX enzyme was measured in homogenate obtained from quinoa seeds. For optimum pH measurement, 0.05 M phosphate buffers with pH 4.0, 4.5, 5.0, 5.5, 6.0, 6.5, 7.0 7.5 and 8.0, 0.05 M Tris-HCl buffers with pH 7.5, 8.0 8.5 and 9.0, 0.05 M Glycine buffer with pH 9.0, 9.5 and 10.0 were prepared. Enzyme activity measurements were performed for each buffer separately. For linoleic acid substrate, homogenate obtained from quinoa seeds was treated with 0.005 M, 0.025 M, 0.05 M, 0.1 M, 0.2 M, 0.4 M, 0.5 M, 0.6 M, 0.8 M, 1.0 M phosphate buffers for LOX enzyme. Activity was measured for each solution.

| 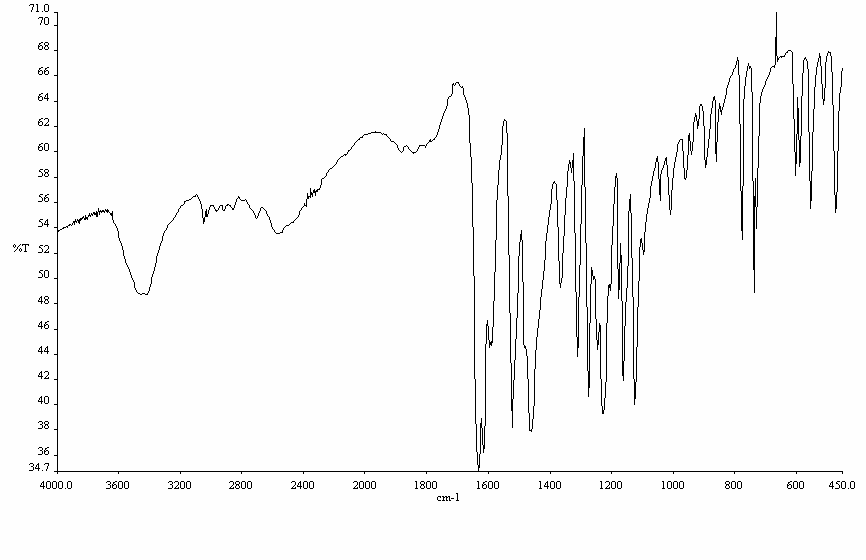 |
| --- |
| 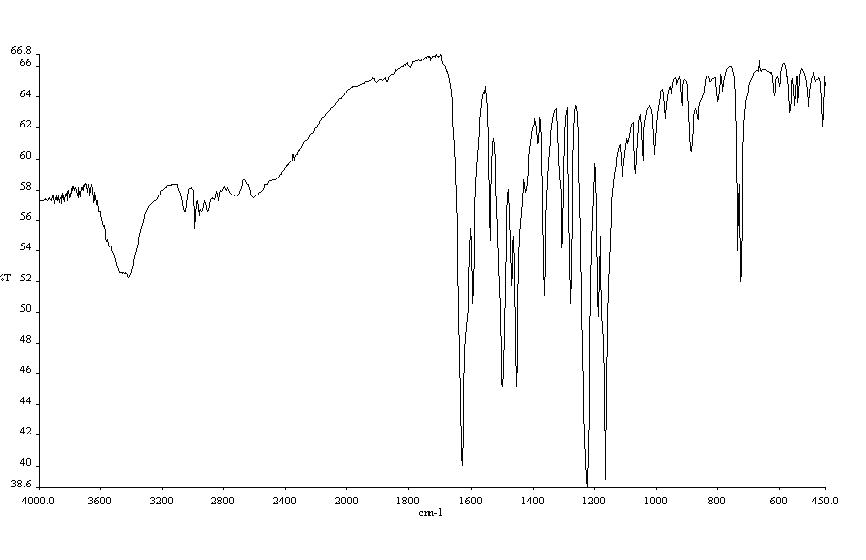 |
| **Figure S1.** IR spectrum of L^1^H (top) and L^2^H (bottom). |

| 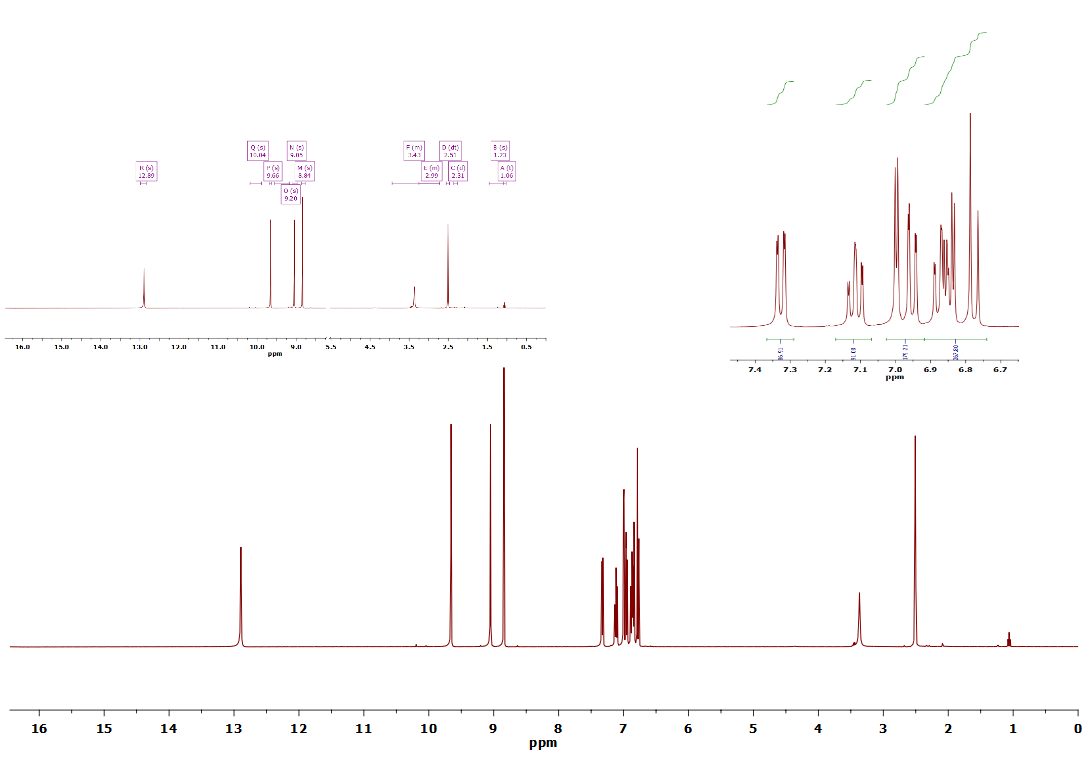 |
| --- |
| 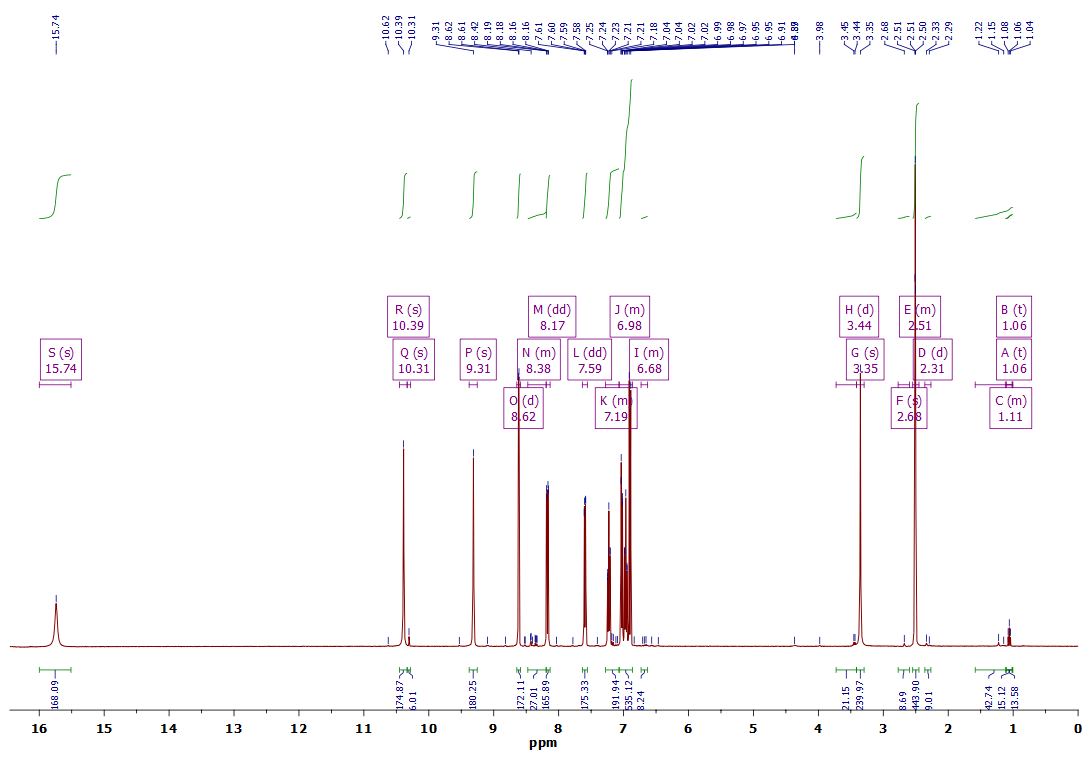 |
| **Figure S2.** ^1^H-NMR spectrum of L^1^H (top) and L^2^H (bottom). |

| _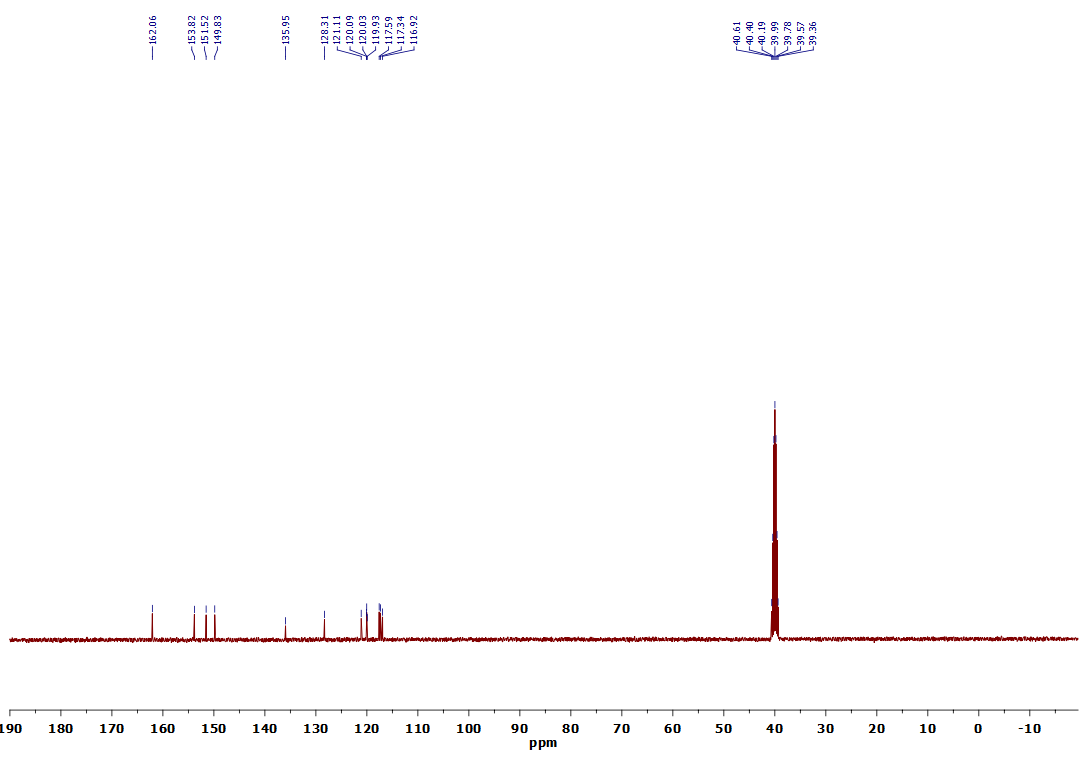_ |
| --- |
| 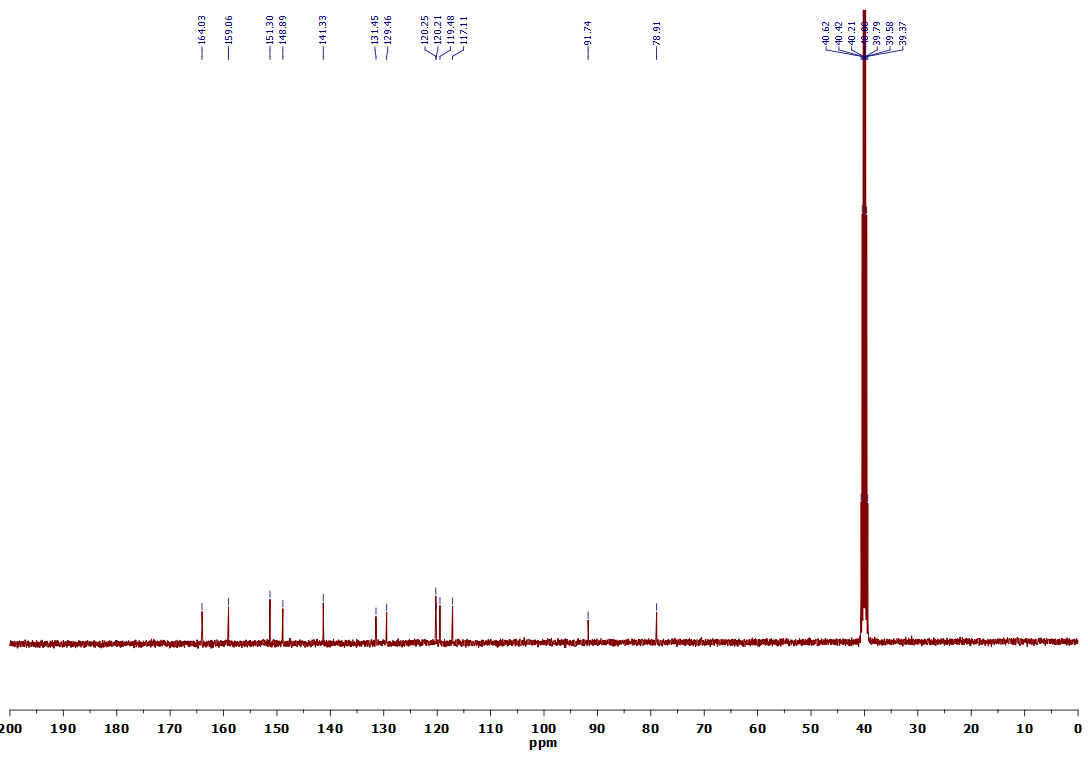 |
| **Figure S3.** ^13^C-NMR spectrum of L^1^H (top) and L^2^H (bottom). |

|  |
| --- |
| **** |
| **Figure S4.** XRD pattern of L^1^H (top) and L^2^H (bottom). |

| 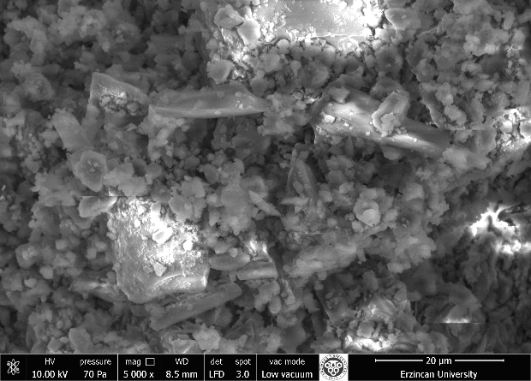 |
| --- |
| 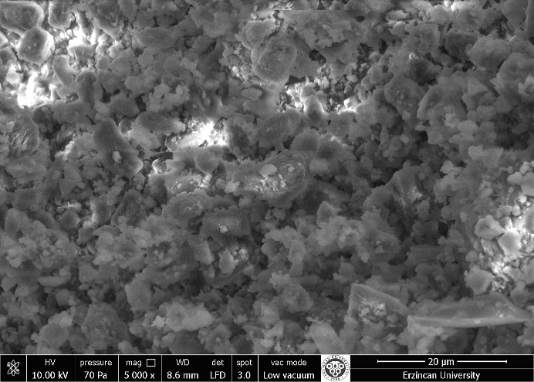 |
| **Figure S5. S**EM of L^1^H (top) and L^2^H (bottom). |

|  |
| --- |
| ****  **** |

**Figure S6.** UV Vis. spectrum of L^1^H and metal complexes (top) and L^2^H and metal complexes (bottom).
